# 546 When Do Patients Arrive to the Burn Center? “Business Hours” Don’t Apply to Burns

**DOI:** 10.1093/jbcr/irae036.180

**Published:** 2024-04-17

**Authors:** Rohit Mittal, Kathleen Hollowed, Jeffrey E Carter, Steven A Kahn

**Affiliations:** MUSC/SJCH, Charleston, SC; MUSC, Charleston, SC; University Medical Center (LSU Health), New Orleans, LA; MUSC/SJCH, Charleston, SC; MUSC, Charleston, SC; University Medical Center (LSU Health), New Orleans, LA; MUSC/SJCH, Charleston, SC; MUSC, Charleston, SC; University Medical Center (LSU Health), New Orleans, LA; MUSC/SJCH, Charleston, SC; MUSC, Charleston, SC; University Medical Center (LSU Health), New Orleans, LA

## Abstract

**Introduction:**

Burn patients can present to a medical facility at any given time. By their nature, one cannot predict when a burn injury will occur. Anecdotal accounts suggest that most burn patients present after “business hours,” however, limited objective data exists on presentation patterns of burn injury. Hospitals tend to have less staffing at night, which can impede care of complex patients. Accurate data is critical for resource allocation and patient centered care. This study seeks to characterize the frequency in which burn patients arrive to the burn center after hours.

**Methods:**

Retrospective arrival times of all patients presenting to the emergency room at two different burn centers in different states were collected over a 1-year period. Business hours were defined as 8AM – 4 PM. Patients arrival times were queried via the institutional electronic medical records over a 1-year period. Total number of patients arriving after hours (4PM – 8AM) were compared between centers with a Fisher’s Exact Test. A secondary time window of 6PM – 8AM was also assessed.

**Results:**

A total of 712 patients presented to the ER at both centers – 315 at center A and 397 at center B. Of these, 214 (67.9%) patients at center A and 227 (57.2%) of patients at center B presented after 4PM and before 8AM. Center A had a significantly higher number of patients arrive at night (p=0.004). Overall, 61.9% of all patients presented to the ER after normal business hours at both centers. On average, 51.1% of all patients presented to the ER in the 6PM – 8AM window at both centers.

**Conclusions:**

This study suggests that the majority (>60%) of patients present to the emergency room with a burn injury come after normal business hours. Even when business hours are extended to 6PM, more than half all patients still present after hours. Paradoxically, most centers have fewer key personnel available at night. These results have potential implications on how resources are dedicated with regards to hospital staffing of multidisciplinary burn team members and research staff for studies with time sensitive enrollment. Further study should investigate patient complexity and care quality while capturing more burn centers with a wider variety of size and geographic distribution.

**Applicability of Research to Practice:**

Understanding patient admission, throughput, and census statistics allows optimization of staffing.

